# Supplementary material for: Insight into the dynamics of APOBEC3G protein in complexes with DNA assessed by high speed AFM
Source: Nanoscale Adv. 2019 Sep 4;1(10):4016–24. doi: 10.1039/c9na00457b (PMC7731963; doi:10.1039/c9na00457b)
Supplement: NA-001-C9NA00457B-s004 [file NA-001-C9NA00457B-s004.pdf]

Electronic Supplementary Material (ESI) for Nanoscale Advances.
This journal is © The Royal Society of Chemistry 2019

Supporting Information

Insight into dynamics of APOBEC3G protein in complexes with DNA assessed by high speed AFM

Yangang Pan[a], Luda S. Shlyakhtenko[a*] and Yuri L. Lyubchenko[a*].

[a] Department of Pharmaceutical Sciences, College of Pharmacy, WSH, University of Nebraska Medical Center, Omaha, Nebraska 68198-6025, USA.

E-mail: ylyubchenko@unmc.edu, E-mail: lshlyakhtenko@unmc.edu.

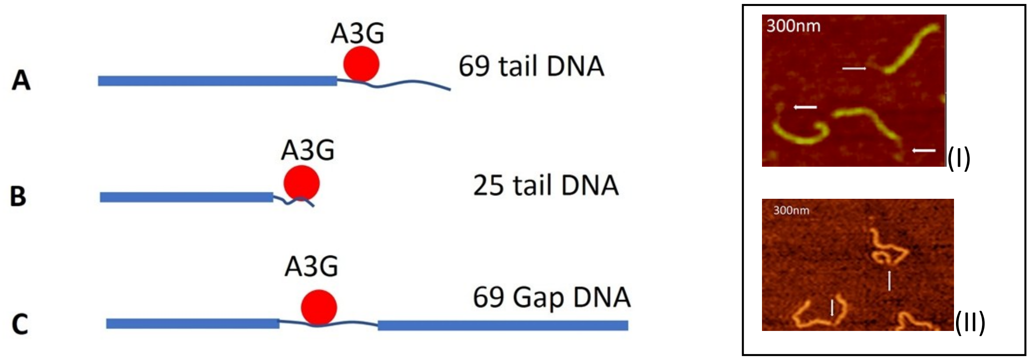

Figure S1. Schematic presentation of tails and gap hybrid DNA constructs. A. The 69 nt tail ssDNA hybrid. **B.** The 25 nt tail ssDNA hybrid. **C.** The 69 nt gap ssDNA hybrid. Thick blue lines correspond to the dsDNA tags used for clear identification of the complexes by AFM. Red balls represent A3G protein attached to the ssDNA part (thin blue line). For tails DNA substrates, A3G appeared as a blob at the end of dsDNA tags. For the A3G-gap DNA complex, A3G is seen in the middle of two dsDNA tags. Inserts i and ii are AFM images of the 69nt tail and gap DNA hybrid respectively. The arrows illustrate the ssDNA parts in the hybrid DNA.

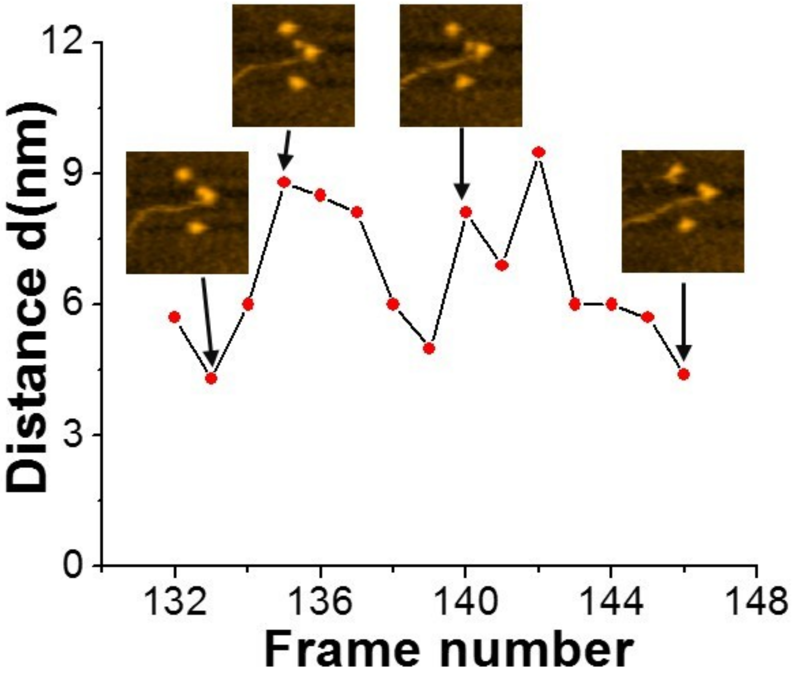

Figure S2. Plot showing the dynamics of the dumbbell structure of A3G in complex with 69 nt ssDNA complex. The arrows point to the maximum and minimum distances between A3G domains, measured from the corresponding AFM frames.

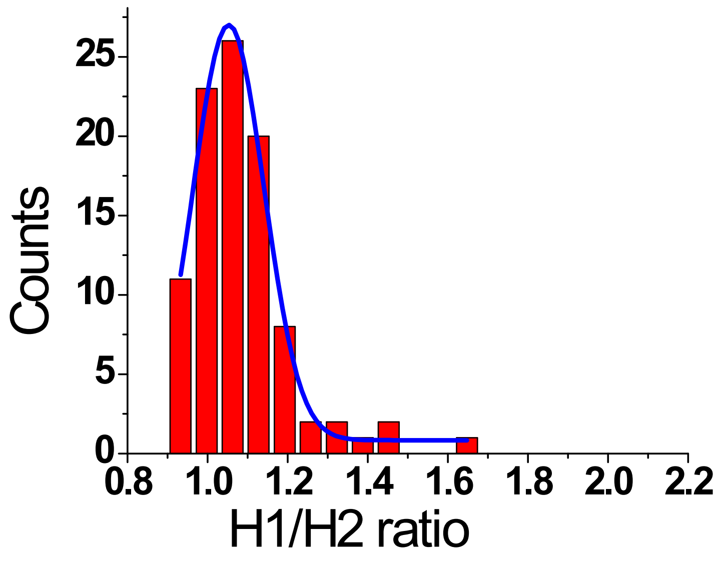

Figure S3. The ratio of heights for Domain 1 (h1) and 2 (h2) for free monomeric A3G.

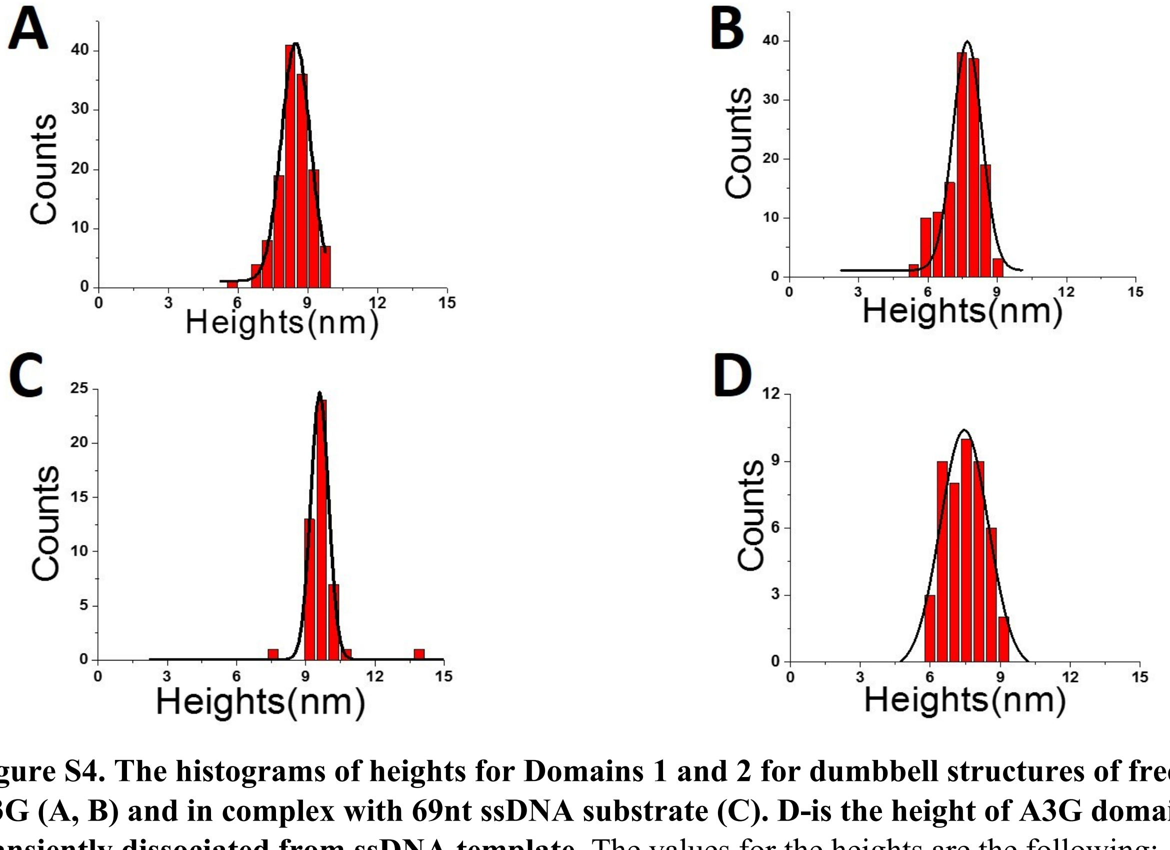

Figure S4. The histograms of heights for Domains 1 and 2 for dumbbell structures of free A3G (A, B) and in complex with 69nt ssDNA substrate (C). D-is the height of A3G domain, transiently dissociated from ssDNA template. The values for the heights are the following: A-

8.4 ±0.6 nm, B- 7.7±0.6 nm, C- 9.6±0.4nm, D- 7.5±1.0nm. All measurements are performed from the same AFM image to exclude possible measurement error.
